# Electrospun PAN/MAPbI_3_ Composite Fibers for Flexible and Broadband Photodetectors

**DOI:** 10.3390/nano9010050

**Published:** 2019-01-02

**Authors:** Gaolin Li, Zhenhua Jiang, Weilin Wang, Zengyong Chu, Ye Zhang, Chunhua Wang

**Affiliations:** College of Liberal Arts and Sciences, National University of Defense Technology, Changsha 410073, China; ligaolin12@nudt.edu.cn (G.L.); wangweilin0930@hotmail.com (W.W.); zhangye8905@foxmail.com (Y.Z.); w_chunhua@tom.com (C.W.)

**Keywords:** perovskite fiber, electrospinning, photodetector, polyacrylonitrile

## Abstract

Methylammonium lead triiodide perovskite (CH_3_NH_3_PbI_3_, MAPbI_3_) has been emerging as an easy processing and benign defect material for optoelectronic devices. Fiber-like perovskite materials are especially in demand for flexible applications. Here we report on a kind of polyacrylonitrile (PAN)/MAPbI_3_ composite fiber, which was electrospun from the mixing solution of PAN and MAPbI_3_. The absorption edge and optical gap of the PAN/MAPbI_3_ composite fibers can be easily tuned as the ratio of the perovskite changes. Both the moisture stability and the thermal stability of the perovskite are improved with the protection of PAN polymers. Flexible photodetectors based on this perovskite fiber were fabricated and analyzed. The photoresponse of the detector was highly sensitive to broadband visible light, and reached 6.5 μA W^−1^ at 700 nm with a voltage bias of 10 V. Compared with pure MAPbI_3_ photodetectors, this composite fiber photodetector has much-improved stability and flexibility, which can even be used to detect motion-related angular changes.

## 1. Introduction

As a new photovoltaic material, methylammonium lead triiodide perovskite (CH_3_NH_3_PbI_3_, denoted as MAPbI_3_) has a low bandgap, a large absorption coefficient, a high carrier mobility, a long carrier diffusion length, and a low-cost synthetic route [1,2,3,4,5,6]. These photoelectric devices, based on organolead halide thin films, are mostly cast from nanocrystals or micro-size grains [7,8,9,10]. Moreover, as a low-dimensional material, pure perovskite fiber has an extremely low defect density, similar to its single crystal counterpart [11,12,13,14], which gives it many potential applications in solar cells, photodetectors, transistors, light-emitting diodes, and so on [15,16]. One-dimensional perovskite fiber is also promising in improving carriers transfer and reducing the recombination of the electron-hole pairs [17,18]. However, the concomitant disadvantage of optoelectronic devices imbedded with fibers is their large surface-area-to-volume ratio [19,20], which usually catalyzes the decomposition of perovskite when exposed to moisture and light, thus degrading the device stability [20,21,22]. As a result, many of these MAPbI_3_ fiber-based photoelectric devices are not satisfactory in performance and stability. Therefore, it was urgent to develop a facile method to prepare MAPbI_3_ fibers with excellent optical trapping and good stability.

Electrospinning is a simple fabrication approach for low-dimensional nanomaterials [23,24,25]. The first patent on electrospinning was reported in the 1930s. From then on, great progress and applications have been made [26,27]. It is simple and convenient for the synthesis of continuous polymeric fibers, and thus also promising for the fabrication of perovskite fibers.

Polyacrylonitrile (PAN) can be co-dissolved with MAPbI_3_ in *N*,*N*-Dimethylformamide solvents, so we chose the composite solution of PAN and MAPbI3 for electrospinning. By performing polymer electrospinning of polyacrylonitrile (PAN) and MAPbI_3_, we successfully synthesized PAN/MAPbI_3_ composite fibers. The obtained fibers exhibit excellent photoelectric and mechanical properties for flexible devices. Photodetectors based on the composite fibers were also constructed and analyzed. These photodetectors exhibit impressive stability and flexible photoresponse under various situations. To our knowledge, this is the first report on perovskite photodetectors based on electrospun PAN/MAPbI_3_ composite fibers. Our work explores a new way towards synthesis of various perovskite/polymer composite materials well-suited for optoelectronic devices with much improved stability.

## 2. Materials and Methods

### 2.1. Synthesis of PAN/MAPbI_3_ Composite Fibers

Chemical grade methyl ammonium iodide (CH_3_NH_3_I), lead iodide (PbI_2_), and *N*,*N*-Dimethylformamide (DMF) were purchased from Xi’an Polymer Light Technology Corp (Xi’an, China). Polyacrylonitrile (PAN) was obtained from Jilin Carbon Fiber Ltd (Jilin, China). All the materials were used as received. The MAPbI_3_ precursor solution was synthesized by mixing CH_3_NH_3_I and PbI_2_ at a 1:1 molar ratio in DMF and stirring for 2 h at 60 °C. The obtained MAPbI_3_ solution is denoted as solution A (2.35 mol/L for MAPbI_3_). The PAN precursor solution was prepared by dissolving polyacrylonitrile in DMF and stirring for 24 h at room temperature. The obtained PAN solution is denoted as solution B (15 wt% for PAN). The PAN/MAPbI_3_ composite solution was prepared by mixing solutions A and B and stirring for 2 h. Here, solution A was added to solution B to make the final mass fraction of PAN change from 15 wt% to 10 wt%, 8 wt%, and 6 wt%.

**Figure 1:**
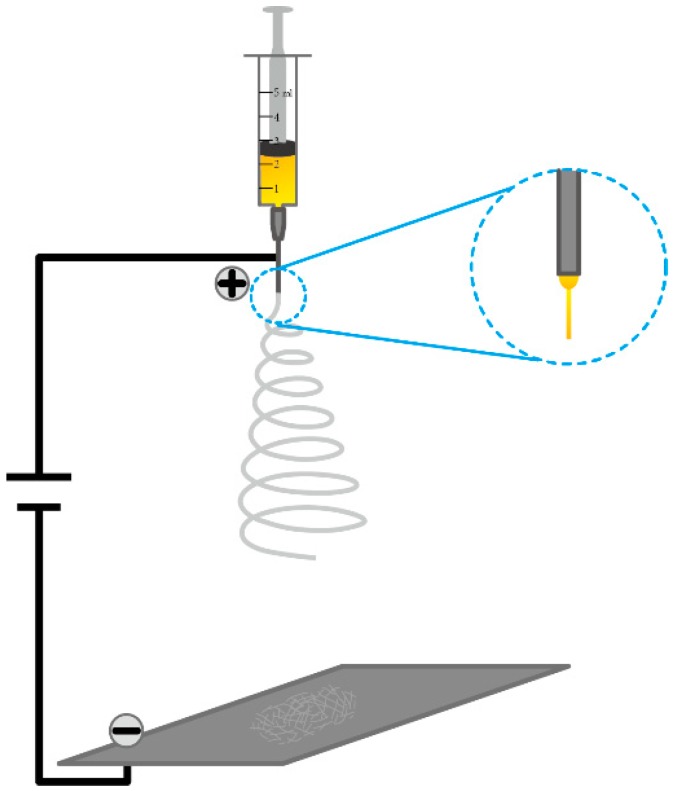
Schematic diagram of the electrospinning setup.

The electrospinning setup is shown in Figure 1, which includes a plastic syringe, an aluminum foil, and DC power. The syringe, with a metallic needle tip, was loaded with the composite precursor solution. The needle tip was connected to the positive electrode (15 kV), and the aluminum foil was connected to the negative electrode. The flow rate was fixed as 1.5 mL/h, and the distance from the needle tip-end to the collector was fixed as 15 cm. The composite fibers deposited on the aluminum foil and formed film under the action of high voltage. Finally, the as-synthesized composite fibers in the film state were dried in an air oven at 65 °C for at least 3 h for removal of the solvent.

The MAPbI_3_ contents of the composite fibers prepared from the 10 wt%, 8 wt%, and 6 wt% solutions were 85.7 wt%, 77.7 wt%, and 66.6 wt% respectively, so the composite fibers were named as M85.7, M77.7, and M66.6, respectively.

### 2.2. Characterization of PAN/MAPbI_3_ Composite Fibers

The surface morphologies of the composite fibers were characterized using a S-4800 scanning electron microscope (SEM) (Hitachi, East Coast Harbor, Honshu Island, Japan). Crystal structures were analyzed with X-ray powder diffraction (XRD), using a Bruker D8ADVANCE diffractometer (Bruker, Karlsruhe, Germany) with Cu-Kα radiation (λ = 1.54178 Å). The crystal distributions of the composite fibers were characterized using a Joel-2100F transmission electron microscope (TEM) (JEOL Ltd., Tokyo, Japan). The ultra-violet-near infra-red (UV-Vis-NIR) diffused reflectance spectrum (DRS) was recorded on a HITACHI U-4100 UV/Vis-NIR spectrophotometer (Hitachi, East Coast Harbor, Honshu Island, Japan). The photoluminescence (PL) spectra were recorded on a HITACHI F-4600 fluorescence spectrofluorometer (Hitachi, East Coast Harbor, Honshu Island, Japan). Thermogravimetric analysis (TGA) was performed using a PerkinElmer STA-6000 thermogravimetric analyzer (PerkinElmer, Waltham, MA, USA).

### 2.3. Fabrication of PAN/MAPbI_3_ Composite Fiber Photodetectors

The photodetector was fabricated in a vertical stack way. From the bottom to the top, the layers were: Glass, PAN/MAPbI_3_ composite fiber film, interdigitated copper electrodes, and glass. The electrodes contacted tightly with the composite fiber film so as to minimize the contact resistance. Notably, the effective area of the photodetectors was as large as 2.40 cm^2^, which is at least one order of magnitude larger than those of other reported perovskite-based photodetectors. Large-area devices have more advantages for practical applications. Flexible photodetectors were constructed through replacing glass substrate with flexible polyethylene terephthalate (PET), which was then coated with silver electrodes. To ensure the accuracy of the results, we assembled five photodetectors for each experiment and carried out photoresponse measurements at least three times for each photodetector. The photoresponses of the photodetectors in this work are the average value of the measurements, which is in a small spread (<0.05 μA).

### 2.4. Photoresponse Measurements

Photoelectronic measurements of the obtained photodetectors were performed in a three electrodes system on an electrochemical workstation (CHI760E). The incident power light was measured by a photoradiometer (PL-MW2000) (PerfectLight, Beijing, China). Different wavelength (420 to 700 nm) lights from a PerfectLight PLS-SXE300/300UV Xenon lamp (PerfectLight, Beijing, China) were focused through filters (Figure S3). Photoelectronic measurements were performed in air and at room temperature without any encapsulation.

## 3. Results and Discussion

### 3.1. Microstructures of the Composite Fibers 

**Figure 2:**
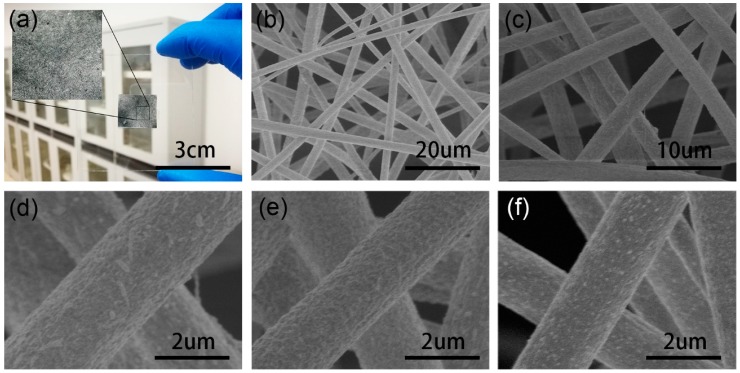
Optical and SEM images of the composite fibers. (**a**) Optical image of the composite fibers. (**b**–**f**) SEM images of the composite fibers: (**b**–**d**) M85.7, (**e**) M77.7, and (**f**) M66.6.

**Figure 3:**
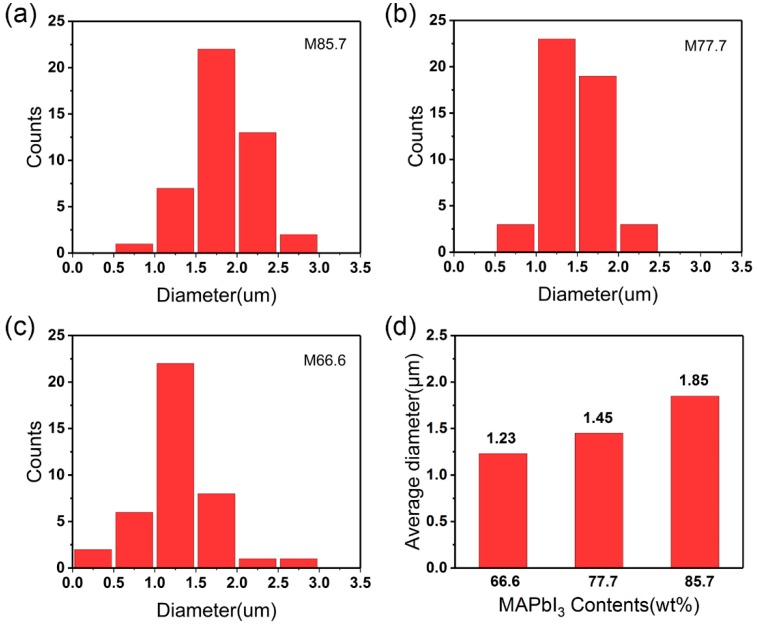
Diameter distributions of the composite fibers. (**a**) M85.7, (**b**) M77.7, and (**c**) M66.6. (**d**) Relationship between the average diameter and MAPbI_3_ content.

The PAN/MAPbI_3_ composite fibers were electrospun from PAN solutions using a setup shown in Figure 1. The fibers were deposited on an aluminum foil in the form of a network film (Figure 2a). Surface morphologies of the composite fibers were characterized using SEM, as shown in Figure 2b–f. The fibers were randomly arranged in nonwoven states. With increases in the content of MAPbI_3_ (e.g., from M66.6 to M85.7), the surfaces of the PAN/MAPbI_3_ composite fibers became rougher, and even rougher than pure PAN fibers (Figure S1). The average diameter of the PAN/MAPbI_3_ composite fibers was about 1–2 μm, and seemed to increase with increasing MAPbI_3_ content (Figure 3).

**Figure 4:**
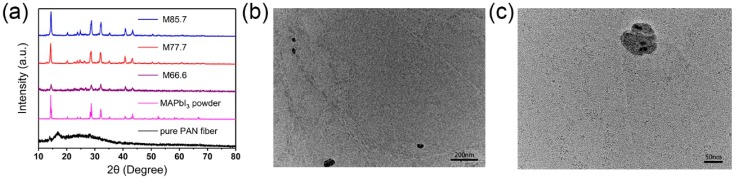
XRD and TEM profiles of the composite fibers. (**a**) XRD of the composite fibers, MAPbI_3_ powder and pure polyacrylonitrile (PAN), and (**b**,**c**) TEM images of the composite fibers.

Corresponding X-ray diffractions of the MAPbI_3_ crystals imbedded in the composite fibers are shown in Figure 4a. The XRD patterns from the reference crystals prepared by bottom seeded solution growth (BSSG) match well with those of the composite fibers. It is noticed that there are some differences in the peak quantity of the composite fibers and pure MAPbI_3_ powders. The composite fibers have several new peaks at 12.95°, 22.19°, and 26.25°, which may correspond to the reflections from the PAN [28]. The XRD results reveal that the synthetic composite fibers are consisting of PAN and the pure phase of MAPbI_3_ with good crystallinity. It is also found that the peak intensities of the composite fibers become stronger with increases in the content of MAPbI_3_. In order to observe the crystal distribution of the composite fibers, composite fibers were grinded and characterized using TEM. As shown in Figure 4b,c, nanocrystals imbedded in the fibers were evenly distributed, which is in agreement with cross-sectional SEM images of the composite fibers (Figure S2).

### 3.2. Optical Property of the Composite Fibers

**Figure 5:**
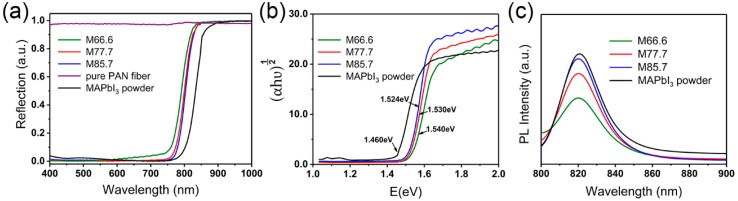
Optical properties of the composite fibers, MAPbI_3_ powder, and pure PAN fibers. (**a**) UV-Vis-NIR diffused reflectance spectrum (DRS), (**b**) bandgap, and (**c**) photoluminescence spectra.

Figure 5a depicts the DRS spectra of pure MAPbI_3_ perovskite powders, PAN/MAPbI_3_ composite fibers, and pure PAN fibers. The white PAN fibers are reflective to visible light, while the PAN/MAPbI_3_ perovskite fibers have a broadband absorption of visible light due to the incorporation of black MAPbI_3_ perovskite nanocrystals. All the reflection spectra of the composite fibers have a clear band edge cutoff, which corresponds to the typical semiconductor absorption. With the increase of perovskite content, there is a red-shift of the band edge cutoff, which gets closer to that of the pure MAPbI_3_ powder. Further analysis was carried out using the transformed Kubelka–Munk method with the UV-vis-NIR DRS data, which suggested that the intrinsic bandgap is 1.460 eV for pure MAPbI_3_ perovskite powder, as shown in Figure 5b. The bandgap of composite fibers is about 1.530 eV, which is approximately 60–80 meV shifted toward the higher energy side.

Figure 5c shows the PL spectra of the composite fibers and the pure MAPbI_3_ perovskite powders. Composite fibers and the pure MAPbI_3_ perovskite powders exhibit obvious PL signals, with similar curve shapes mainly located in the near-infrared region. Slight red-shifting of the PL peaks was observed for the composite fibers with the increase of MAPbI_3_ content, which is in accordance with their narrower band gap.

Obviously, the apparent optical band gap of the composite fibers can be slightly tuned by changing the blend ratio of the constituent MAPbI_3_ perovskite, which could broaden the applications of the PAN/MAPbI_3_ composite fibers in the semiconductor device field.

### 3.3. Photovoltaic Performance of the Composite Fibers

**Figure 6:**
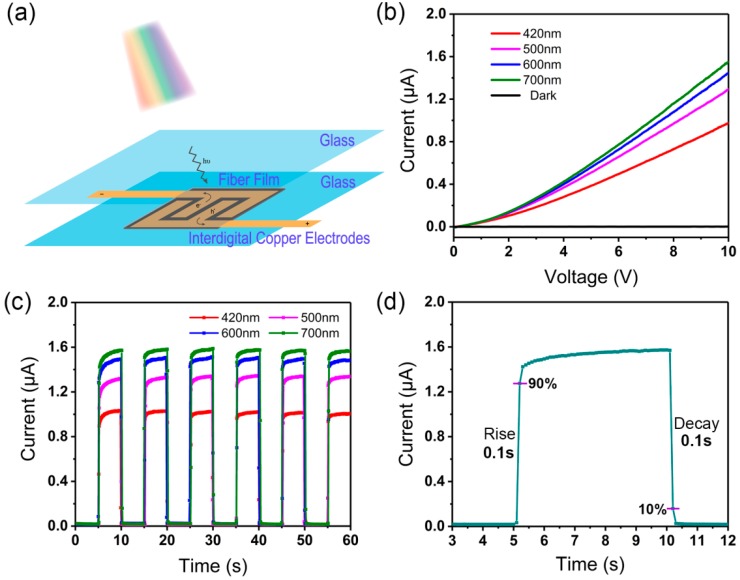
Photoresponse performance of the photodetectors. (**a**) Schematic illustration of the photodetector. (**b**) Current-voltage curve and (**c**) photocurrent responses of the detector based on the M85.7 fibers. (**d**) Transient response of the photodetector based on the M85.7 fibers.

Based on the obtained PAN/MAPbI_3_ composite fibers, photodetectors were constructed on two pieces of glass substrates, as illustrated in Figure 6a. To examine the photoelectric properties of the composite fiber photodetectors, the photoresponses were characterized under simulated sunlight. For a conductive photodetector, the responsivity (*R*) and external quantum efficiency (*EQE*) are the figures of merit to evaluate the performance of a different geometry device. *R* and *EQE* are calculated by the following equations:

(1) $$R=(I_{light}-I_{dark})/E_{light}$$

(2) EQE=hcR/eλ

where *I_light_* and *I_dark_* are the current under light and dark respectively, *E_light_* is the power of incident illumination, *h* is the Planck constant, *c* is the speed of light, *λ* is the wavelength of the incident light, and *e* is the unit charge.

The photoresponse was measured at different wavelengths, ranging from 420 to 500, 600, and 700 nm (Figure S3). As shown in Figure 6b, different photocurrent responses under different wavelengths were observed for the photodetector based on M85.7 composite fibers. Under the light illumination of 100 mW cm^−2^, the photocurrent of the device increased gradually from 1.0 to 1.5 μA, with an increase in the light wavelength from 420 to 700 nm, which is attributed to an increase in the quantity of electrons excited. This is agreeable with other reported perovskite photodetectors [9,10,11,12,13,14,15,16]. The photoconduction is based on the electron-hole pairs, which are excited due to the incident light with energy larger than the band gap. The conductance of the device can be increased significantly with light illumination. Although a photon with larger energy in the shorter wavelength will excite more electrons [6], a beam of longer-wavelength light has more photons under the same light intensity (100 mW cm^−2^). Therefore, the transition probability increases for longer wavelengths at the same light intensity, resulting in a large increase in free-carrier density and easier carrier transport and injection, thus enhancing the photocurrent of the photoelectric devices greatly, which was reflected in the increase of the photocurrent (1.5 μA).

The photoresponse was measured as a function of time during repetitive switching of different-wavelength light illumination at a bias of 10 V, as shown in Figure 6c. The saturated photocurrent increased with increasing of the light wavelength from 420 to 700 nm, which is consistent with the current-voltage (CV) curve results. For the M85.7 composite fiber, the responsivity *R* reached 6.5 μA W^−1^ at 700 nm, with an extremely low *EQE* (0.002%). The *EQE* of these photodetectors is poor, mainly because the carrier diffusion length is much shorter than the distance between two neighboring fingers (0.75 mm). Consequently, a large number of pairs vanished because of the enhanced charge recombination [29]. The composite fiber photodetector could be rapidly and reversibly switched on and off, periodically at approximately 5-s intervals. When the Xenon lamp was switched on, a fast-transient current could be observed. In addition, the undeniable role of the quality of the composite fibers is also indicated by the time dependence of the photocurrent. As shown in Figure 6d, the rise time was measured as the transition time between the minimum current to 90% of the maximum value, and the fall time was defined from the maximum value down to 10%. For the M85.7 photodetector, the characteristic rise and fall times were both approaching 0.1 s. It is reasonable to suppose that an increase in MAPbI_3_ perovskite content could increase the responsivity.

**Figure 7:**
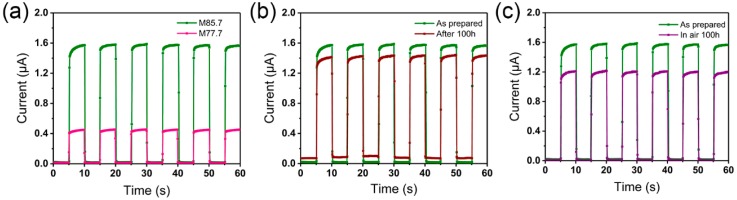
Photoresponse performance of the photodetectors. (**a**) Comparison of photocurrent between M77.7 and M85.7 at 700 nm. (**b**) M85.7 device retained over 85% of its initial responsivity after 100 h exposure to moisture. (**c**) M85.7 device retained nearly 75% of its initial responsivity after fibers were exposed to ambient air for 100 h.

The content of perovskite plays a critical role in the performance of the photodetectors, as shown in Figure 7a. The M85.7 photodetector had the strongest photocurrent response under the same conditions, due to its higher perovskite content. The on/off current ratio of the M85.7 photodetector could achieve around 100 at 420 nm, and 80 at 700 nm at a bias of 10 V. The dark current was only 10^−8^ A. However, the M77.7 photodetector could only achieve an on/off current ratio of 23 at 700 nm.

Upon Xenon lamp illumination, plenty of electron-hole pairs can be generated in the hybrid perovskite, and excellent contact can be formed between the copper electrodes and the composite fibers. Therefore, the higher the perovskite content in the composite fibers, the stronger the photocurrent of the photodetectors. The low *I_dark_* indicates an impressively low free-carrier density. The migration of electrons and holes is inhibited because of the high-contact barrier at the interface of Cu/perovskite in the dark. Therefore, the photodetectors can reach a high photo-to-dark current ratio at 10 V, indicating that the composite fibers are suitable for practical photon-detection applications.

All these results demonstrate that PAN/MAPbI_3_ composite fibers can be used in fast photoelectric switches and highly sensitive photodetectors. Compared to other MAPbI_3_ fiber photodetectors, which have been reported to be unstable in the majority of ordinary solvents and the air environment, the composite perovskite fiber photodetectors not only show remarkable responses, but also have much better stability.

As shown in Figure 7b, the stability and photoresponses of the composite fiber photodetectors were studied after 100 h exposure to moisture. The devices retained over 85% of their initial responsivity, without deterioration of the key photoelectric properties. This suggests that the polymer may act as a protection buffer between MAPbI_3_ and atmospheric moisture, elongating the lifetime of the photodetectors in ambient air. Predictably, M66.6 and M77.7 have better stability because of their higher content of PAN. M85.7 composite fibers were placed in air firstly for 100 h, and then used to fabricate photodetectors. Nearly 75% of their initial responsivity was retained, as shown in Figure 7c. Their reduced stability was due to the lack of the protection of glass encapsulation when exposed to air, indicating that glass encapsulation is another important factor that influences the stability. 

**Figure 8:**
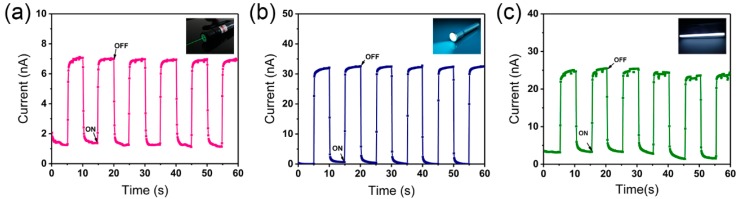
Photoresponse performance under different light sources. (**a**) Laser, (**b**) flashlight, and (**c**) fluorescent lamp.

As shown in Figure 8a–c, these photodetectors also exhibit impressive photoresponses under various conditions (laser, flashlight, and fluorescent lamp), and maintain their performance after 100 h exposure in air.

### 3.4. Thermal Stability of the Composite Fibers 

**Figure 9:**
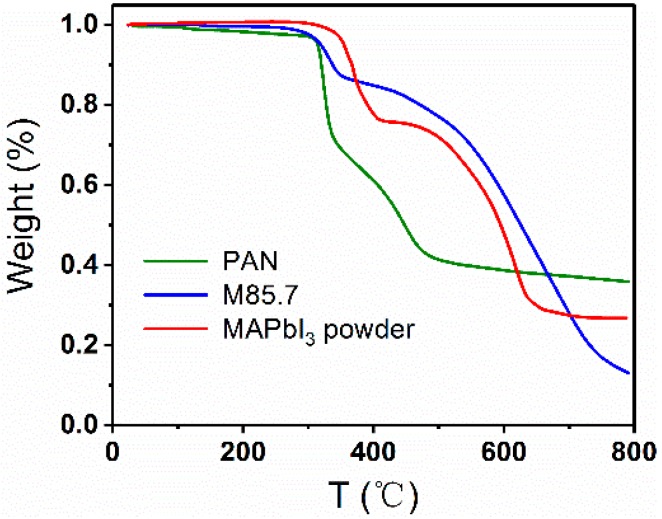
TGA curves of the M85.7 fibers, PAN fibers, and pure MAPbI_3_ powder.

The thermal stability is also an important figure of merit to evaluate the application of the composite fibers. TGA measurements were carried out with a heating rate of 20 °C/min under N_2_ atmosphere, and the results are shown in Figure 9. MAPbI_3_ is generally thermally stable up to 300 °C [4]. The M85.7 composite fibers start losing slight weight at 160 °C. The curve indicates that the thermal decomposition of the as-spun fibers is completed in three distinct steps. In the first step, the weight loss is observed between 160 and 300 °C, which may be due to the evaporation of DMF. In the second and third steps (300–400 and >400 °C), the weight loss of approximately 70 wt% is due to the degradation of MAPbI_3_ and PAN. The composite fibers are thermally stable and fully meet the design and work requirements of the photoelectric devices.

### 3.5. Motion Detections of the Flexible Photodetectors

**Figure 10:**
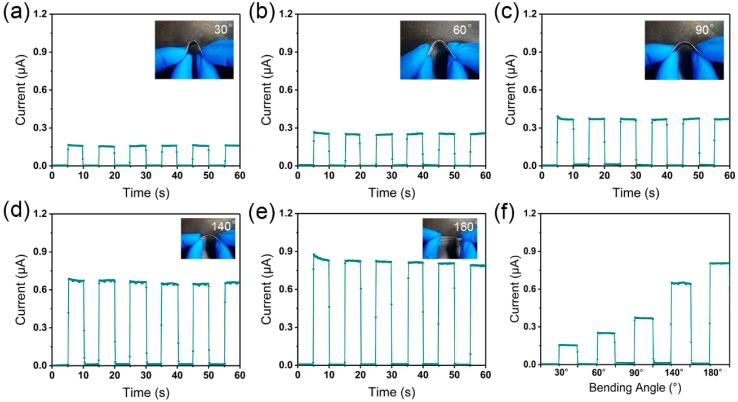
Motion detections of the flexible photodetectors. (**a**–**e**) Photocurrent of flexible composite fiber photodetectors under different bending angles. (**f**) Relationship between photocurrent and bending angles.

Flexible photodetectors were also constructed through replacing glass substrate with flexible polyethylene terephthalate (PET), which was then coated with silver electrodes. To examine the relationship between photocurrent and flexibility of the photodetectors, the photoresponse was characterized under different bending angles, as shown in Figure 10. As the bending degree decreased, the photocurrent became stronger. The flexible photodetector showed the strongest photocurrent response without bending. This result demonstrates that bending angles play a critical role in the photocurrent of the flexible photodetectors. Therefore, this flexible detector may be used to detect motion-related angular changes (Figure 10f).

## 4. Conclusions

In summary, electrospun PAN/MAPbI_3_ perovskite fibers were successfully fabricated, and broadband photodetectors were constructed from the obtained composite fibers. MAPbI_3_ perovskite nanocrystals were detected in the composite fibers, which have much-improved stability due to the protection of PAN polymers. It is also possible to adjust the absorption edge or the bandgap of the composite fibers by controlling the content of MAPbI_3_. The photodetectors have high sensitivity, rapid photoresponses, and improved stability. The flexible photodetectors based on the obtained M85.7 composite fibers show different photocurrent responses at different bending angles. These studies suggest that a simple fabrication of perovskite/polymer composite fibers by electrospinning can broaden the applications of perovskite in the optoelectronic fields, and inspire the development and application of different polymer/perovskite composite systems. Our strategy may pave the way towards polymer/perovskite composite fibers suitable for various flexible photoelectronic devices.

## Supplementary Materials

The following are available online at http://www.mdpi.com/2079-4991/9/1/50/s1, Figure S1: SEM images of polyacrylonitrile (PAN) fibers, Figure S2: SEM cross-section images of the composite fibers, Figure S3: Transmission of different wavelength filters.
