# Supplementary material for: Electrospun PAN/MAPbI3 Composite Fibers for Flexible and Broadband Photodetectors
Source: Nanomaterials (Basel). 2019 Jan 2;9(1):50. doi: 10.3390/nano9010050 (PMC6358830; doi:10.3390/nano9010050)
Supplement: Supplementary file 1 [file nanomaterials-09-00050-s001.pdf]

nanomaterials

Supporting Information

Electrospun PAN/$MAPbI_3$ Composite Fibers for Flexible and Broadband Photodetectors

Gaolin Li, Zhenhua Jiang *, Weilin Wang, Zengyong Chu *, Zhang Ye and Chunhua Wang

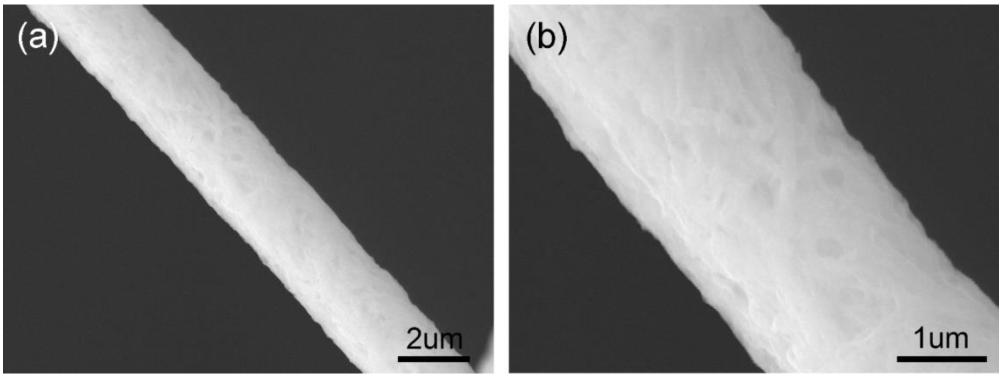

Figure S1. SEM images of polyacrylonitrile (PAN) fibers.

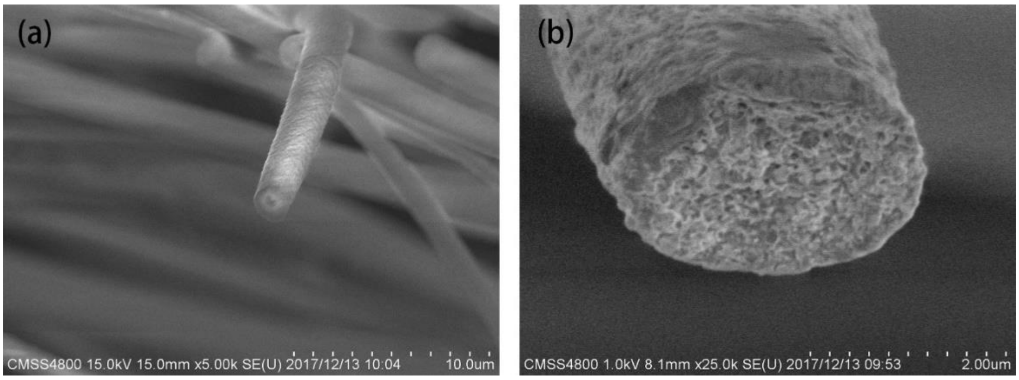

Figure S2. SEM cross-section images of the composite fibers.

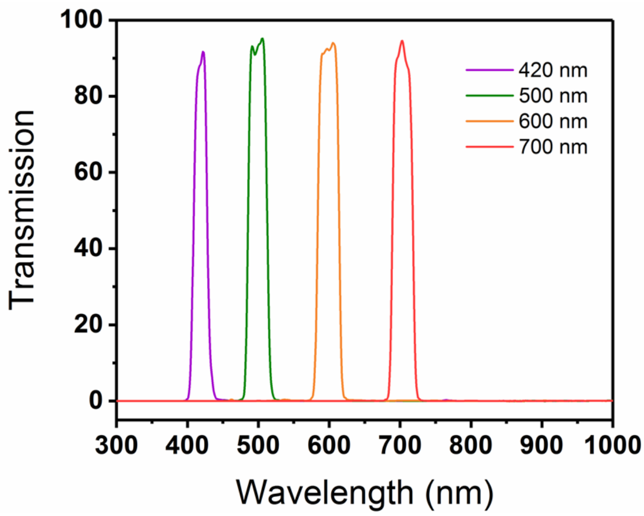

Figure S3. Transmission of different wavelength filters.
